# Using internet search queries for infectious disease surveillance: screening diseases for suitability

**DOI:** 10.1186/s12879-014-0690-1

**Published:** 2014-12-31

**Authors:** Gabriel J Milinovich, Simon M R Avril, Archie C A Clements, John S Brownstein, Shilu Tong, Wenbiao Hu

**Affiliations:** School of Public Health and Social Work, Queensland University of Technology, Brisbane, Australia; Infectious Disease Epidemiology Unit, School of Population Health, The University of Queensland, Brisbane, Australia; Freelance developer, Bundaberg, Australia; Research School of Population Health, ANU College of Medicine, Biology and Environment, The Australian National University, Canberra, Australia; Department of Pediatrics, Harvard Medical School and Children’s Hospital Informatics Program, Boston Children’s Hospital, Boston, USA

## Abstract

**Background:**

Internet-based surveillance systems provide a novel approach to monitoring infectious diseases. Surveillance systems built on internet data are economically, logistically and epidemiologically appealing and have shown significant promise. The potential for these systems has increased with increased internet availability and shifts in health-related information seeking behaviour. This approach to monitoring infectious diseases has, however, only been applied to single or small groups of select diseases. This study aims to systematically investigate the potential for developing surveillance and early warning systems using internet search data, for a wide range of infectious diseases.

**Methods:**

Official notifications for 64 infectious diseases in Australia were downloaded and correlated with frequencies for 164 internet search terms for the period 2009–13 using Spearman’s rank correlations. Time series cross correlations were performed to assess the potential for search terms to be used in construction of early warning systems.

**Results:**

Notifications for 17 infectious diseases (26.6%) were found to be significantly correlated with a selected search term. The use of internet metrics as a means of surveillance has not previously been described for 12 (70.6%) of these diseases. The majority of diseases identified were vaccine-preventable, vector-borne or sexually transmissible; cross correlations, however, indicated that vector-borne and vaccine preventable diseases are best suited for development of early warning systems.

**Conclusions:**

The findings of this study suggest that internet-based surveillance systems have broader applicability to monitoring infectious diseases than has previously been recognised. Furthermore, internet-based surveillance systems have a potential role in forecasting emerging infectious disease events, especially for vaccine-preventable and vector-borne diseases.

**Electronic supplementary material:**

The online version of this article (doi:10.1186/s12879-014-0690-1) contains supplementary material, which is available to authorized users.

## Background

Prudent detection is a cornerstone in the control and prevention of infectious diseases. Traditional infectious disease surveillance systems are typically characterised by a bottom-up process of data collection and information flow; these systems require a patient to recognise illness and seek treatment and a physician or laboratory to diagnose the infection and notify the relevant authority [1],[2]. For emerging infectious disease events, this process is reported to take, on average, 15 days from onset to detection and a further 12–24 hours for the World Health Organization to be notified [3]. The development and implementation of more efficient systems for gathering intelligence on infectious diseases has the potential to reduce the impact of disease events. Internet-based surveillance systems are one such system [4].

Internet-based surveillance systems produce estimates of disease incidence through analysis of various digital data-sources. Targeted sources include internet-search metrics, online news stories, social network data and blog/microblog data [4]. Currently, the most promising approach appears to be those based upon monitoring of internet search behaviour. This approach works on the premise that people will actively seek information on diseases they develop and that estimates of disease activity with the community may be developed by monitoring the frequency of related internet searches. Through targeting people earlier in the disease process, internet-based systems are able to access a larger fraction of the community and produce more timely information. Furthermore, internet-based surveillance systems are intuitive and adaptable, cheap to run and maintain (once established), do not require a formal public health network and have the capacity to be automated and operate in near-real time. Despite these advantages, internet-based surveillance systems have a number of significant shortcomings and must not be considered an alternative to traditional surveillance approaches [5]. Firstly, as these systems crowd-source data, resolution will be contingent on the size of the population serviced and may be further limited by national communications infrastructure availability and distribution [6]. Secondly, as internet-based surveillance systems are limited to people who use the internet to source health information, there is the potential that estimates produced by these systems may not accurately reflect the entire community [7]. Finally, as internet-based surveillance systems essentially rely upon self-reporting, bias may be introduced through differences in internet usage between sectors of the community (the elderly, for example, may not use the internet as a source of health information, despite being a high-risk group for many infectious diseases) and/or through media driven interest in emerging disease events [4].

Infectious diseases surveillance systems have been developed using internet search metrics to estimate incidence of influenza (Google Flu Trends) [8] and dengue (Google Dengue Trends) [9]. Currently, operational systems that utilise this approach are limited, however, studies of the potential for internet-based surveillance have been conducted for a range of other infectious diseases, including: acute respiratory illness [7], AIDS [10], chickenpox [11],[12], cryptosporidiosis [13], dysentery [10], gastroenteritis [11], Hepatitis [14], listeriosis [15], Lyme disease [16], methicillin-resistant *Staphylococcus aureus* [17], norovirus [18], respiratory syncytial virus [6], rotavirus [19], scarlet fever (*Streptococcus pyogenes*) [10],[20], *Salmonella* [21], tuberculosis [10],[22] and West Nile virus [6]. Previous studies have focused on single diseases, or a small number of diseases, and the justification of the focus on a particular disease has been specific to each study. The published results have largely been promising; however, to date there has been no systematic, generalizable analysis to identifying diseases that are suited to monitoring through the analysis of internet-search metrics.

The underpinning goal of this study was to provide direction for future approaches to developing digital surveillance systems; such as the development of predictive models and/or integrative surveillance models that draw upon multiple traditional and digital data source to create estimates of disease within the community. This study, however, did not aim to develop actionable surveillance systems, produce predictive models of infectious disease based on internet-based data or to identify the best search terms for use in these models. Rather, this study aimed to determine which diseases have most promise for monitoring by surveillance systems built on internet search metrics; this was achieved by assessing the level of correlation between a wide range of infectious diseases and internet search term metrics. Finally, this study aims to identify diseases for which internet-based data could be used to create early warning systems.

## Methods

### Infectious disease surveillance data

Surveillance data on notifiable infectious diseases were collected from the National Notifiable Disease Surveillance System (NNDSS) which is maintained by the Australia Government Department of Health (DoH) [23]. Monthly notifications (case numbers) aggregated at state/territory and national level, were downloaded for the period of January 2004 to September 2013. A full list of notifiable diseases in Australia and case definitions can be accessed through the DoH webpage [24]. Sixty-four diseases are monitored and these are categorised in the NNDSS as belonging to one of eight groups: blood-borne diseases; gastrointestinal diseases; other bacterial diseases; quarantinable diseases; sexually transmissible infections; vector-borne diseases; vaccine preventable diseases; and zoonoses. For the purpose of consistency, we have reported diseases according to these groupings. Whilst notifiable, data were not downloaded for human immunodeficiency virus infection/acquired immunodeficiency syndrome, Creutzfeldt–Jakob disease or variant Creutzfeldt–Jakob disease because surveillance for these diseases is not performed by DoH or for severe acute respiratory syndrome, because reporting to the DoH is informal; as such, these diseases are not listed on the NNDSS.

### Search term selection and scraping of internet search trend data

In the construction of Google Flu Trends model, the authors identified search terms by performing correlations between influenza-like illness data from the US CDC and the top 50 million Google search queries performed in the US over the corresponding period [8]. Such data is not available to the public and an alternative approach to identification of search terms was required; two approaches were used. Firstly terms related to diseases, the aetiological agents and colloquialisms (such as “hep” for hepatitis or “flu” for influenza) were manually identified. Secondly, Google Correlate (www.google.com/trends/correlate) was queried using monthly surveillance data (described above). Google Correlate provides a list of up to 100 search terms that correlate most highly with the query data. To account for potential language shifts that may have affected search behaviour [4], this was performed three times using surveillance data covering the periods 2004–13, 2007–13 and 2011–13. Up to 300 search terms were downloaded from Google Correlate for each notifiable disease (100 search terms per period analysed) and manually sorted; any term related to the queried notifiable disease was included, regardless of the nature of the potential association Suitable terms were combined with the manually identified search terms to create a list of search terms (see Additional file 1). No attempt was made to filter search terms based upon biological plausibility; any term that may be perceived to have any association with the disease of interest was included.

Search frequencies for terms of interest were collected through Google Trends (www.google.com/trends/). All data extractions were performed on the 22nd of October, 2013. Google Trends was queried using each of the identified terms at a national and state/territory level using the entire time range available (2004–present). Google Trends presents search frequency as a normalised data series with values ranging from 0 to 100 (with 100 representing the point with the highest search frequency and other points scaled accordingly); functionality for exporting search frequency data as a .CSV file is provided. For the purpose of privacy, data are aggregated at a daily, weekly or monthly level (or are restricted if there is insufficient search volume). The level of aggregation applied is determined by the period analysed and the search frequency; the level of aggregation is not able to be specified by the user. As the notifiable disease surveillance data used was in monthly format, monthly indices of query search frequencies were required. Monthly indices are displayed graphically by Google Trends when querying periods greater than 36 months; rather than downloading.CSV files, a script was developed to scrape data from the Google Trends webpage, allowing the problems associated with the level of data aggregation to be overcome.

### Data analysis

Analyses were performed at both national and state levels for the period 2009–13. As state-level search frequency data were not always available, particularly for less common diseases (due to low search frequency at this level of disaggregation), correlations between state-level notification data and national search frequency data were also performed. Owing to the large number of correlations performed in this study, Bonferroni adjustments [25] were applied to significance levels by the equation 1-(1-α)^1/n^; all *p*-values reported in this document correspond to one-tailed tests. Spearman’s rank correlation coefficients were used to rank performance.

Time-series cross correlations were performed to assess linear associations between disease notifications and Google Trend search indices. Cross correlations were calculated using lag values for Google Trends data ranging from −7 to 7. This range allowed for assessment of biologically plausible associations that were relevant to the development of early warning systems. Cross correlations were performed on national data using IBM SPSS version 21 (SPSS Inc; Chicago, IL, USA). Seasonal differencing was applied (value 1) to all analyses to remove cyclic trends.

**Figure 1:**
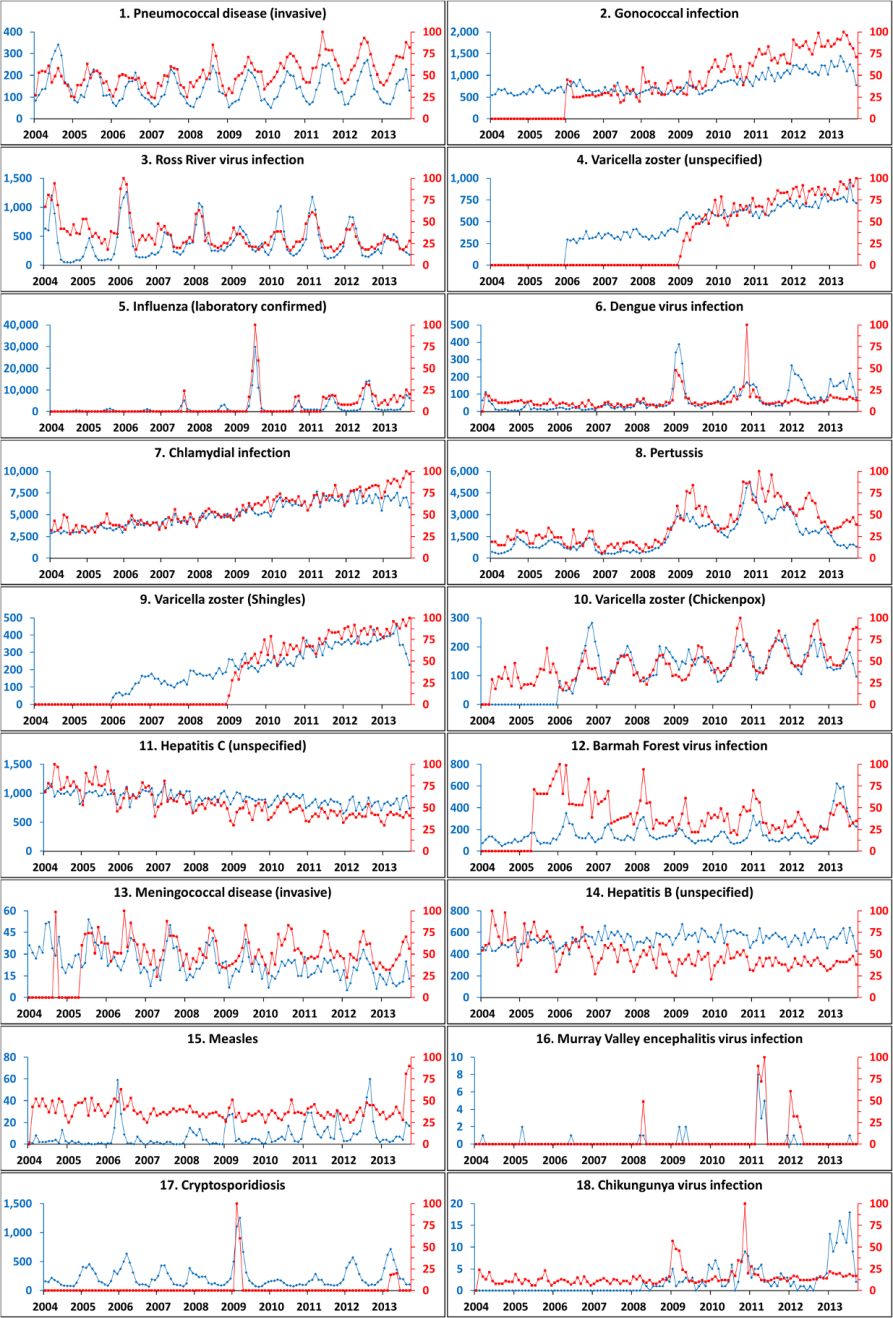
**Top internet search terms analysed for 18 diseases with the highest Spearman’s rho values (2009–13).** National monthly case numbers (blue) and Australian Google Trend search index (red). Google Trend search terms used in the analysis are presented in Figure 2.

Whilst all available data (2004–13) were downloaded, analyses for this study were focused on the most recent five years (2009–13) as preliminary data analyses indicated that Google Trends data were not available prior to 2009 for numerous search terms (Figure 1; panels 2, 4, 9, 12, 16 and 17). Additionally, shifts in language are known to affect surveillance systems built upon textual data [4]. The shortened period (2009–13) was selected to minimise the effects of language shifts. However, this period still provides the requisite 50 pairs of observations for performing cross correlations [26].

## Results

In this section we discuss analyses of time series data. Briefly, the time series analysed were monthly case numbers for the 64 infectious diseases monitored by the Australian Government’s National Notifiable Disease Surveillance System (NNDSS) and Google Trends monthly search metrics for related internet search terms. In total, search 164 terms were analysed in this study; this ranged from a single term for some diseases, up to 14 search terms for influenza and 35 search terms for pneumococcal disease. The majority of terms could be categorised as diseases or aetiological agents (“brucellosis” or “Brucella”), colloquialisms (“flu”, “hep” or “TB”), symptoms (“cough”, “white discharge” or “cervical mucus”) or medication or general health/treatment related queries (“whooping cough treatment”, “symptoms of dengue” or “flu and pregnancy”). A few terms that may have environmental (“flash floods” for leptospirosis) or behavioural (“African tours” for malaria) meanings were also included. A full list of the search terms analysed is presented in the supplementary material.

### Spearman’s correlations

**Figure 2:**
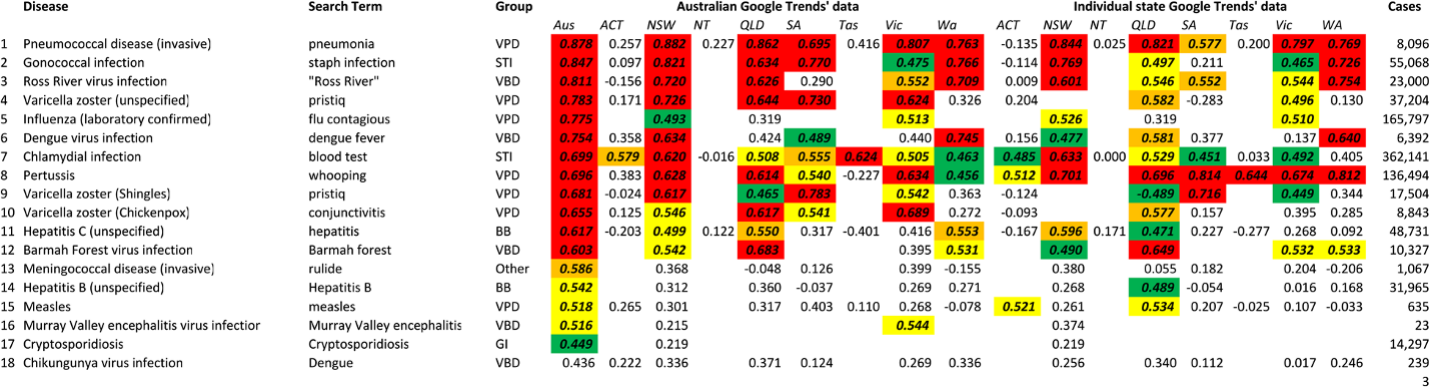
**Spearman’s rho values for the 18 top ranked notifiable diseases for the period 2009–13.** The table only contains the search term with the highest degree of correlation for each disease; see Additional file 1 for a full list of diseases, search terms and correlation coefficients. The column label in bold indicates the Google Trends data used and subheadings in italics indicate the disease notification data used. Case numbers are National totals for the period 2009–13. Shading denoted statistical significance (one-tailed, Bonferroni corrected) at 0.0001 (red), 0.001 (orange), 0.01 (yellow) and 0.05 (green) levels. For disease grouping, BB: Blood-borne diseases; GI: Gastrointestinal diseases; Other; Other bacterial diseases; QD; Quarantinable diseases; STI: Sexually Transmissible Infections; VBD: Vector-borne Diseases; VPD: Vaccine preventable diseases; Zoo: Zoonoses.

Evaluation of the bivariate associations between surveillance and corresponding search frequency data was performed using the Spearman’s rank correlation. Spearman’s rank correlations for the 18 top ranked notifiable diseases and terms are presented in Figure 2 and raw data for the corresponding diseases and search terms are presented in Figure 1. Results of Spearman’s correlations indicated 17 diseases to be significantly correlated (p < 0.05; Bonferroni corrected: p < 2.43E^−04^) with at least one search term; *p*-values for 12 of these were <0.0001 (Bonferroni corrected: p < 4.74E^−07^). Marked differences were observed in correlations between the various disease groups. Correlations for vaccine-preventable diseases were generally highest with six of fourteen exhibiting strong (rho =0.60-0.799) or very strong (rho =0.80-1.00) correlations, followed by sexually transmitted infections (2/6), the vector-borne diseases (3/9), blood-borne diseases (1/6), other diseases (1/4), zoonoses (0/8), gastrointestinal infections (0/11) and, finally, quarantinable diseases (0/6). State level correlations are also reported in Figure 2. Consistency between state correlations were variable with some diseases exhibiting reasonable consistency (pertussis; rank 8), whilst others were inconsistent (hepatitis C; rank 11).

### Cross correlations

**Figure 3:**
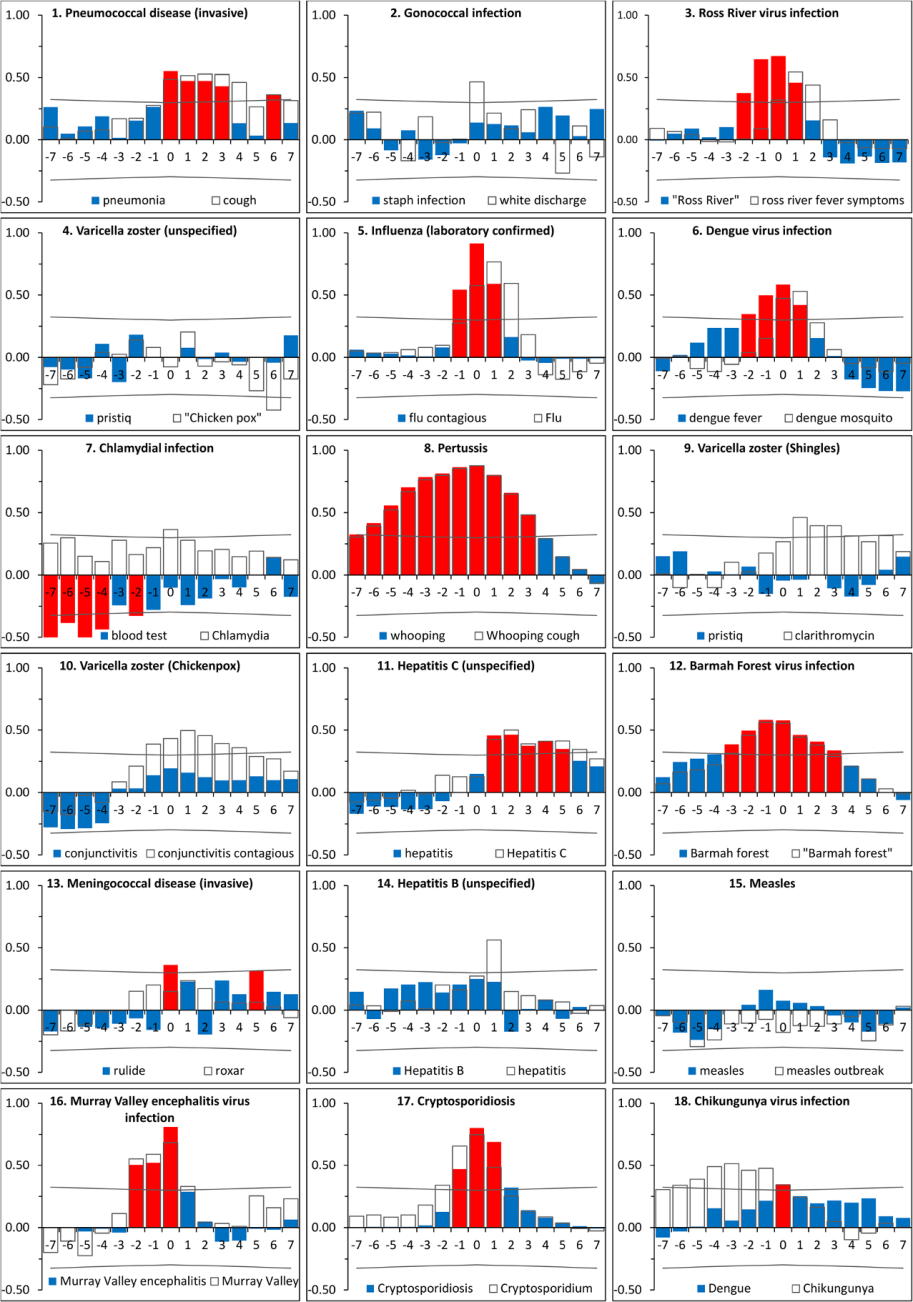
**Cross correlation results for the 18 diseases with the highest Spearman’s rho values (2009–13).** Cross correlations for two search terms are displayed for each disease. Coloured bars correspond to the search term with the highest Spearman’s rho value for each disease (red bars indicate values that exceed the 95% confidence interval, whereas blue bars do not). Unfilled bars indicate cross correlation results for alternative search terms with highest cross correlation values at a lag value of 1. Confidence intervals (95%) are indicated by the grey lines.

Results of cross correlations are demonstrated in Figure 3. Cross correlation results should be interpreted as product–moment correlations between the two time series; they allow dependence between two time series to be identified over a series of temporal offsets, referred to as lags. Lag values indicate the degree and direction of associations. A lag value of −1 indicates that correlations were performed using time series data for which the first series (Google Trends’ data) has been shifted backwards one unit (a month). Conversely, a lag value of 1 indicates that the primary series had been shifted forward one unit. Significant positive correlations for lag vales of ≥1 or above are of most interest in the context of this study as they indicate a positive relationship between the two time series with Google Trends data leading the notifications (a pre-requisite for Google Trends data to be a suitable early warning tool). It should also be noted that seasonal differencing was applied to cross correlations to remove cyclic seasonal trends.

Disease notifications positively correlated at a lag of one month (lag 1) with search term frequency for 12 of the 17 diseases that exhibited significant Spearman’s rank correlations. Overall, 15 of the 64 notifiable diseases exhibited significant, positive correlations at lag of one month. Significant positive associations were observed for four of the nine vector-borne diseases (Barmah Forest virus infection, Dengue virus infection, Murray Valley encephalitis virus infection and Ross River virus infection), six of the 14 vaccine preventable diseases (*Haemophilus influenzae* type b, influenza, pertussis, pneumococcal disease and varicella zoster (chickenpox and shingles)), two of the six blood-borne diseases (hepatitis B (unspecified) and C (unspecified)), two of 11 gastrointestinal diseases (campylobacteriosis and cryptosporidiosis) and one zoonosis (leptospirosis). Positive significant correlations were not observed at a lag of one month for any of the quarantinable diseases (n = 6), sexually transmissible infections (n = 6) or other bacterial infections (n = 4). It should be noted that positive significant correlations were observed at lags of over one month (but not at lag 1) for two of the top ranked 18 diseases (gonococcal infection and meningococcal disease) and 16 diseases overall (see Additional file 1). Additionally, the terms “haemolytic uraemic syndrome” and “leprosy” exhibited significant negative correlations with the respective disease notifications at a lag of one month.

## Discussion

The development and application of internet-based infectious disease surveillance systems has the potential to enhance infectious disease control and prevention. Whilst this is widely recognised [4],[6],[7],[12],[15],[16],[18],[20] the investigation and application of internet-based surveillance has not been systematically applied across infectious diseases; the lack of systemic knowledge regarding the potential breadth of internet-based surveillance appears to have restricted the development of systems to a small number of diseases. To our knowledge, assessments of the use of internet-based surveillance have only been performed for five of the 17 diseases that were demonstrated to have a significant association with internet search terms (influenza [4], dengue [9],[27], chickenpox [11],[12], hepatitis B [14] and cryptosporidiosis [13] – the authors of the final study were, however, not able to detect signals from internet search queries). Our study suggests that internet-based surveillance systems have potential application to a wider range of diseases than is currently recognised. However, correlations alone should not be viewed as definitive evidence that such systems are viable; some discretion must be applied, particularly as the analyses performed were univariate. Correlations between internet metrics and both gonococcal infection and chlamydia (Figure 1, boxes 2 and 7) were high; this appears to be due to a general upward trend in both and internet metrics appears to have little value in detecting perturbations in cases beyond this. This is supported by the cross correlation results (which are seasonally differenced); despite being ranked 2nd and 7th by Spearman rho (Figure 2), no positive correlations were observed for these disease/search term cross correlations, even at lag 0 (Figure 3). Further research needs to be performed; however, this study suggests surveillance systems build on internet search data to have significant promise for a number of diseases beyond those previously described, most notably pneumococcal disease, Ross River virus infection, pertussis, Barmah Forest virus and invasive meningococcal disease.

The application of internet-based data to monitoring systems of interest has been termed “nowcasting”; this approach does not predict the occurrence of future events, but rather seeks to produce more timely information on the systems of interest [28]. For infectious disease surveillance, this is typically achieved through the ability of internet-based surveillance systems to collect data at an earlier time point than is possible for traditional systems or by circumventing bureaucratic structures inherent to traditional systems that impede information flow [4]. Search terms that exhibit a high level of correlation with disease notifications are of value as they may be used to provide faster intelligence on emerging disease events. Results of cross correlations (Figure 3), however, indicated that forecasting of infectious disease events may also be possible using internet-based data. Of the 17 diseases that exhibited significant Spearman’s correlations, 12 also had significant positive cross correlations at a lag of one month. Overall, cross correlations indicated that forecasting of notification rates using internet-based metrics would be most realistic for the vaccine-preventable and vector-borne diseases. Despite search terms offering strong or very strong correlations for two of the sexually transmissible diseases, neither exhibited significant correlations at a lag of one month.

Whilst internet metrics may provide valuable information regarding disease status, it is important to view these within context. The term “dengue mosquito” (Figure 3, panel 6) leads notifications by up to one month. The data imply dependence of dengue notifications on searches for the term “dengue mosquito”. The mechanism of this dependence is more likely that environmental conditions that increase the abundance of mosquitos in dengue risk areas correlate with both an increase in dengue notifications and increased search interest for “dengue mosquito”, allowing the search term to be used as an indicator for notifications. In this context the internet metrics also provide information that is of potential significance with respect to control of dengue fever; there is increased interest regarding mosquitos in the community and this may be driven by an increase in mosquito numbers. Conversely the incidence of disease in the community may also affect search habits. The search term “chikungunya” lags notifications for chikungunya virus infection (Figure 3, panel 18). Searches for “chikungunya” are probably driven by media exposure. Media bias has previously been reported to adversely affect internet-based surveillance systems [27],[29]-[33] and an increase in cases of a disease in the community will likely result in the publication of stories about the disease in the media; in turn, media exposure will drive internet searches on the topic. These processes, however, are not necessarily mutually exclusive. Searches for a disease may lead notifications, however, increased notifications and reporting of an emerging disease event in the media may also drive internet searches. The complexity of this relationship may make interpretation of Google Trends’ data more difficult. For pertussis (Figure 3, panel 8), the term “whooping” exhibits a significant positive correlation with disease notifications from lag −7 through to lag 3. It appears that both mechanisms occur for the same term, demonstrating a potential difficulty in interpreting these data. It is imperative that any terms used in the development of forecasting models are heavily screened to address the complexities of the driving forces behind health-information seeking and routinely re-evaluated to account for any shifts in search behaviour which may occur [4].

There were a number of obvious limitations to this study. The temporal resolution of the data used was monthly. Internet-based surveillance systems built upon monthly data are unlikely to provide better intelligence than existing traditional surveillance systems; these commonly rely upon weekly or daily reporting. This was a function of the availability of the notification data. Secondly, the analyses were performed for a specific setting: Australia. The nuances of language will create differences in the applicability, not just for different countries, but also within a country and between different settings (such as during an influenza pandemic) [4]. Australia was selected as the study area because internet penetration in Australia is very high (>80%) [34] and use is largely restricted to a single search engine; Google maintains a market share of over 90% in Australia [35]. These features reduce biases associated with unequal patterns of use and/or access. Additionally, owing to its extensive size, Australia exhibits a range of climates and varying environmental conditions, making it susceptible to a wide range of infectious diseases, including endemic and non-endemic vector-borne diseases. Additionally, Australia has a strong public health network and comprehensive infectious disease surveillance systems which compile high quality data on a range of diseases. Combined, these features of internet usage and availability, infectious disease surveillance systems and diseases susceptibility patterns make Australia an ideal system in which to study the potential application of internet-based surveillance systems. It is hoped that this work will stimulate further research into internet-based infectious disease surveillance systems beyond Australia. Even within our own study, however, we observed variation in correlations between internet search metrics and disease notifications for the various states (Figure 2). It is imperative to develop models specific to the region of interest and to assess the performance of any internet-based system against traditional surveillance data specific to the region being monitored. Thirdly, this study analysed the performance of only single search terms in estimating infectious disease notifications. Whilst Google has not revealed the terms utilised, or the weightings applied, Google Flu Trends is reported to incorporate around 160 search terms [36]. Despite using only a single search term for each analysis, notifications for 13 diseases were identified as having a strong or very strong correlation with the selected search terms. Compounding this is the fact that Bonferroni adjustments were applied in assessing significance. Bonferroni adjustments have previously been criticised for being overly conservative and for increasing the occurrence of type II errors (false negatives) [25]. As such, whilst this study provides a base for future research, it would be remiss to limit future investigations to just these diseases.

This study identified numerous infectious diseases of public health significance that had not previously been investigated to have potential for monitoring using internet-based surveillance systems However, this study did not seek to produce robust, accurate, internet-based surveillance systems or early warning systems that are able to produce actionable and timely data for public health units. The aim of this study was to identify the diseases for which this is possible and to focus future research efforts into these. To achieve this aim, this study used univariate analyses to determine the usefulness of internet search metrics for monitoring a wide range of infectious diseases. Whilst this simplistic approach was useful for screening diseases, it will not suffice in monitoring or forecasting incidence. Future studies should focus on developing composite indexes incorporate multiple search terms, or data sources (such as weather data). Models built in such a manner are more resilient to media-driven behaviour, fear-based searching and evolutions in language [4]. Internet-based surveillance systems have the potential to be applied to more than just enumerating disease cases within the community or predicting the onset, peak and magnitude of outbreaks. Internet-based systems also have value as tools for planning emergency department staffing and surge capacity [31],[37] or for healthcare utilisation [38]. Future research needs to also investigate to application of internet-based data; the greatest challenge in this field may not actually be creating models for forecasting or monitoring disease within the community, but rather applying and articulating the significance in a manner that is beneficial.

## Conclusions

Internet-based surveillance systems have broader applicability for the monitoring of infectious diseases than is currently recognised. Furthermore, internet-based surveillance systems have a potential role in forecasting of emerging infectious disease events.

## Additional file

## Electronic supplementary material

Additional file 1: Complete tables of results for Google Correlate Searches, Google Trends data, Spearman Correlations and cross correlations. (XLSX 2 MB)

Below are the links to the authors’ original submitted files for images.

Authors’ original file for figure 1

Authors’ original file for figure 2

Authors’ original file for figure 3

## References

[1] Castillo-Salgado C (2010). Trends and directions of global public health surveillance. Epidemiol Rev.

[2] Zeng X, Wagner M (2002). Modeling the effects of epidemics on routinely collected data. J Am Med Inform Assoc.

[3] Chan EH, Brewer TF, Madoff LC, Pollack MP, Sonricker AL, Keller M, Freifeld CC, Blench M, Mawudeku A, Brownstein JS (2010). Global capacity for emerging infectious disease detection. Proc Natl Acad Sci U S A.

[4] Milinovich GJ, Williams GM, Clements ACA, Hu W (2014). Internet-based surveillance systems for monitoring emerging infectious diseases. Lancet Infect Dis.

[5] Lazer D, Kennedy R, King G, Vespignani A (2014). Big data. The parable of Google Flu: traps in big data analysis. Science.

[6] Carneiro HA, Mylonakis E (2009). Google trends: a web-based tool for real-time surveillance of disease outbreaks. Clin Infect Dis.

[7] Valdivia A, Lopez-Alcalde J, Vicente M, Pichiule M, Ruiz M, Ordobas M: Monitoring influenza activity in Europe with Google Flu Trends: comparison with the findings of sentinel physician networks - results for 2009–10. *Euro surveillance: bulletin europeen sur les maladies transmissibles = European communicable disease bulletin* 2010, 15(29):pii=19621., 10.2807/ese.15.29.19621-en 20667303

[8] Ginsberg J, Mohebbi MH, Patel RS, Brammer L, Smolinski MS, Brilliant L (2009). Detecting influenza epidemics using search engine query data. Nature.

[9] Chan EH, Sahai V, Conrad C, Brownstein JS (2011). Using web search query data to monitor dengue epidemics: a new model for neglected tropical disease surveillance. PLoS Negl Trop Dis.

[10] Zhou XC, Shen HB (2010). Notifiable infectious disease surveillance with data collected by search engine. J Zhejiang Univ-SCI C.

[11] Pelat C, Turbelin C, Bar-Hen A, Flahault A, Valleron A (2009). More diseases tracked by using Google trends. Emerg Infect Dis.

[12] Valdivia A, Monge-Corella S (2010). Diseases tracked by using Google trends, Spain. Emerg Infect Dis.

[13] Andersson T, Bjelkmar P, Hulth A, Lindh J, Stenmark S, Widerstrom M (2014). Syndromic surveillance for local outbreak detection and awareness: evaluating outbreak signals of acute gastroenteritis in telephone triage, web-based queries and over-the-counter pharmacy sales. Epidemiol Infect.

[14] Zhou X, Li Q, Zhu Z, Zhao H, Tang H, Feng Y (2013). Monitoring epidemic alert levels by analyzing internet search volume. IEEE Trans Biomed Eng.

[15] Wilson K, Brownstein JS (2009). Early detection of disease outbreaks using the internet. Can Med Assoc J.

[16] Seifter A, Schwarzwalder A, Geis K, Aucott J (2010). The utility of "Google trends" for epidemiological research: Lyme disease as an example. Geospat Health.

[17] Dukic VM, David MZ, Lauderdale DS (2011). Internet queries and methicillin-resistant staphylococcus aureus surveillance. Emerg Infect Dis.

[18] Desai R, Hall AJ, Lopman BA, Shimshoni Y, Rennick M, Efron N, Matias Y, Patel MM, Parashar UD (2012). Norovirus disease surveillance using Google internet query share data. Clin Infect Dis.

[19] Desai R, Lopman BA, Shimshoni Y, Harris JP, Patel MM, Parashar UD (2012). Use of internet search data to monitor impact of rotavirus vaccination in the United States. Clin Infect Dis.

[20] Samaras L, Garcia-Barriocanal E, Sicilia MA (2012). Syndromic surveillance models using Web data: the case of scarlet fever in the UK. Inform Health Soc Care.

[21] Brownstein JS, Freifeld CC, Madoff LC (2009). Digital disease detection–harnessing the Web for public health surveillance. N Engl J Med.

[22] Zhou X, Ye J, Feng Y (2011). Tuberculosis surveillance by analyzing Google trends. IEEE Trans Biomed Eng.

[23] National Notifiable Diseases Surveillance System. [http://www9.health.gov.au/cda/source/cda-index.cfm]

[24] Australian national notifiable diseases and case definitions. [http://www.health.gov.au/internet/main/publishing.nsf/Content/cdna-casedefinitions.htm]

[25] Perneger TV (1998). What’s wrong with Bonferroni adjustments. BMJ: British Medical Journal.

[26] Box GE, Jenkins GM, Reinsel GC (2008). Time Series Analysis: Forecasting and Control.

[27] Althouse BM, Ng YY, Cummings DA (2011). Prediction of dengue incidence using search query surveillance. PLoS Negl Trop Dis.

[28] Choi HY, Varian H (2012). Predicting the present with Google trends. Econ Rec.

[29] Hulth A, Rydevik G: Web query-based surveillance in Sweden during the influenza A(H1N1)2009 pandemic, April 2009 to February 2010. *Euro surveillance: bulletin europeen sur les maladies transmissibles = European communicable disease bulletin* 2011, 16(18):pii=19856., 21586265

[30] Ortiz JR, Zhou H, Shay DK, Neuzil KM, Fowlkes AL, Goss CH (2011). Monitoring influenza activity in the United States: a comparison of traditional surveillance systems with Google Flu trends. PLoS One.

[31] Dugas AF, Hsieh YH, Levin SR, Pines JM, Mareiniss DP, Mohareb A, Gaydos CA, Perl TM, Rothman RE (2012). Google Flu trends: correlation with emergency department influenza rates and crowding metrics. Clin Infect Dis.

[32] Watts G (2008). Google watches over flu. BMJ (Clinical research ed).

[33] McDonnell WM, Nelson DS, Schunk JE (2012). Should we fear "flu fear" itself? Effects of H1N1 influenza fear on ED use. Am J Emerg Med.

[34] World Telecommunication/ICT Indicators Database 2013 (17th Edition). [http://www.itu.int/en/ITU-D/Statistics/Pages/publications/wtid.aspx]

[35] StatCounter Global Stats - Top 5 seach engines in Australia from 2008 to 2013. [http://gs.statcounter.com/#search_engine-AU-yearly-2008-2013]

[36] Cook S, Conrad C, Fowlkes AL, Mohebbi MH (2011). Assessing Google flu trends performance in the United States during the 2009 influenza virus A (H1N1) pandemic. PLoS One.

[37] Araz OM, Bentley D, Muelleman R: Using Google Flu Trends Data in Forecasting Influenza-Like–Illness Related Emergency Department Visits in Omaha, Nebraska. *The American journal of emergency medicine* 2014, In Press., 10.1016/j.ajem.2014.05.052 25037278

[38] Schuster NM, Rogers MA, McMahon LF (2010). Using search engine query data to track pharmaceutical utilization: a study of statins. Am J Manag Care.

