# Supplementary figures and images for: Using internet search queries for infectious disease surveillance: screening diseases for suitability
Source: BMC Infect Dis. 2014 Dec 31;14:690. doi: 10.1186/s12879-014-0690-1 (PMC4300155; doi:10.1186/s12879-014-0690-1)

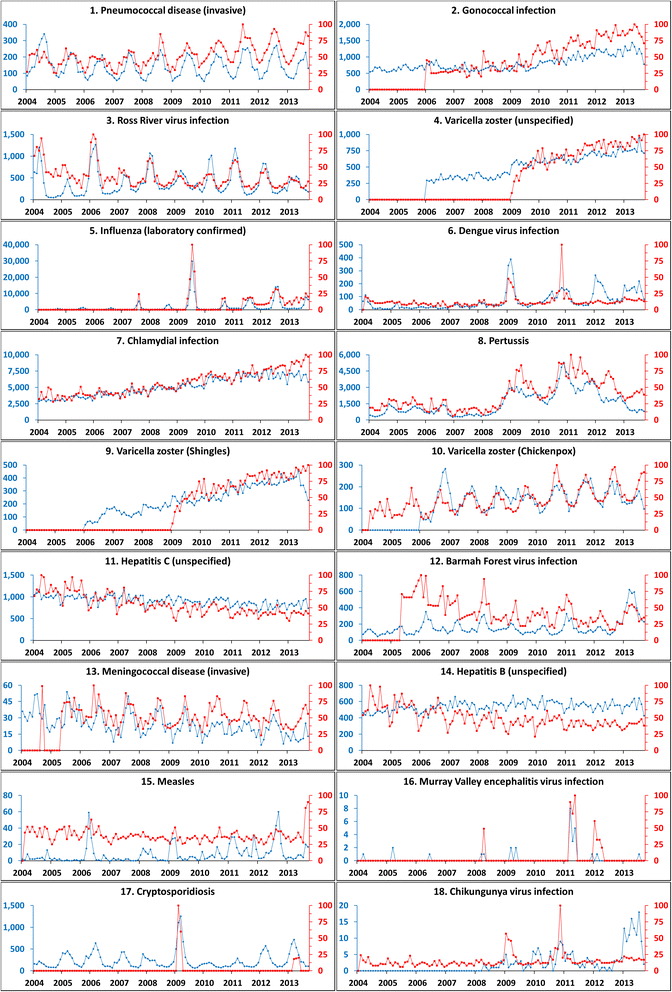

Supplement: Supplementary file 2 — Authors’ original file for figure 1 [file 12879_2014_690_MOESM2_ESM.gif]

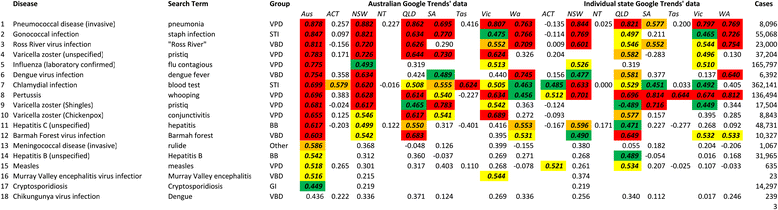

Supplement: Supplementary file 3 — Authors’ original file for figure 2 [file 12879_2014_690_MOESM3_ESM.gif]

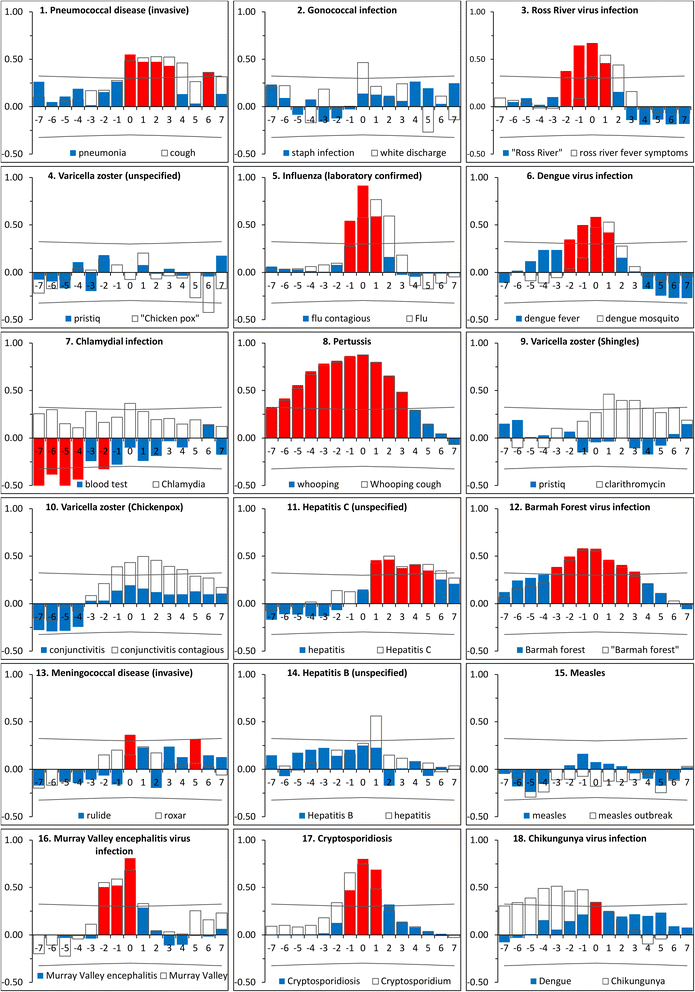

Supplement: Supplementary file 4 — Authors’ original file for figure 3 [file 12879_2014_690_MOESM4_ESM.gif]
